# Direct Lymph Node Vaccination of Lentivector/Prostate-Specific Antigen is Safe and Generates Tissue-Specific Responses in Rhesus Macaques

**DOI:** 10.3390/biomedicines4010006

**Published:** 2016-02-19

**Authors:** Bryan C. Au, Chyan-Jang Lee, Orlay Lopez-Perez, Warren Foltz, Tania C. Felizardo, James C.M. Wang, Ju Huang, Xin Fan, Melissa Madden, Alyssa Goldstein, David A. Jaffray, Badru Moloo, J. Andrea McCart, Jeffrey A. Medin

**Affiliations:** 1 University Health Network (UHN), Toronto, ON M5G 2C4, Canada; bryan.cy.au@gmail.com (B.C.A.); chyanjang@gmail.com (C.-J.L.); orlaylopezpe@yahoo.ca (O.L.-P.); tania.felizardo@nih.gov (T.C.F.); jamescm.wang@mail.utoronto.ca (J.C.M.W.); juhuang@uhnresearch.ca (J.H.); fancy0828@hotmail.com (X.F.); amccart@uhnres.utoronto.ca (J.A.M.); 2 Radiation Medicine Program, Princess Margaret Hospital, UHN, Toronto, ON M5G 2M9, Canada; Warren.Foltz@rmp.uhn.on.ca (W.F.); david.jaffray@rmp.uhn.on.ca (D.A.J.); 3 Animal Resources Centre, UHN, Toronto, ON M5G 1L7, Canada; drmadden@my-vet.ca (M.M.); agoldste@uhnresearch.ca (A.G.); bmoloo@uhnres.utoronto.ca (B.M.)

**Keywords:** preclinical, lentivector, immunotherapy, cancer vaccine, prostate cancer

## Abstract

Anti-cancer immunotherapy is emerging from a nadir and demonstrating tangible benefits to patients. A variety of approaches are now employed. We are invoking antigen (Ag)-specific responses through direct injections of recombinant lentivectors (LVs) that encode sequences for tumor-associated antigens into multiple lymph nodes to optimize immune presentation/stimulation. Here we first demonstrate the effectiveness and antigen-specificity of this approach in mice challenged with prostate-specific antigen (PSA)-expressing tumor cells. Next we tested the safety and efficacy of this approach in two cohorts of rhesus macaques as a prelude to a clinical trial application. Our vector encodes the cDNA for rhesus macaque PSA and a rhesus macaque cell surface marker to facilitate vector titering and tracking. We utilized two independent injection schemas demarcated by the timing of LV administration. In both cohorts we observed marked tissue-specific responses as measured by clinical evaluations and magnetic resonance imaging of the prostate gland. Tissue-specific responses were sustained for up to six months—the end-point of the study. Control animals immunized against an irrelevant Ag were unaffected. We did not observe vector spread in test or control animals or perturbations of systemic immune parameters. This approach thus offers an “off-the-shelf” anti-cancer vaccine that could be made at large scale and injected into patients—even on an out-patient basis.

## 1. Introduction

Recently, several novel technologies have demonstrated impressive clinical efficacies in cancer immunotherapy, including chimeric antigen receptors (CARs) that redirect T cells to specific antigens (Ags) [1], and PD-1 or CTLA-4 blockades that prevent T cell inactivation [2]. Immunotherapy has the potential to target rapidly proliferating cancer, overcome disseminated disease, and even eradicate residual cancer stem cells. However, potent and durable immune responses against tumors are often limited by a vast array of immunosuppressive mechanisms and natural mechanisms of self-tolerance that normally function to prevent autoimmune responses [3,4]. An immunization that could specifically overcome such tolerance to an endogenous tumor-associated antigen (TAA), for example, could lead to beneficial immune responses against cancer. It would be especially useful if such a vaccination could be formulated as an “off-the-shelf” product for ease of delivery and could be readily engineered to target a variety of cancers.

Prostate-reactive lymphocytes seem to be present in prostate cancer (PCa) as evidenced by infiltrating Ag-specific CD4^+^ and CD8^+^ T cells [5,6] and prostate-specific auto-antibodies in PCa patients [7]. Encouragingly, the presence of prostate-infiltrating lymphocytes appears to be associated with improved prognosis [8,9], suggesting the possibility of amplifying pre-existing immune responses to improve clinical outcomes. Sipuleucel-T (Provenge) was recently approved by the FDA as the first therapy aimed at augmenting immune responses for treatment of castration-resistant PCa. Sipuleucel-T consists of autologous Ag-presenting cells (APCs) matured and pulsed *ex vivo* with prostatic acid phosphatase (PAP) Ag fused with the cytokine GM-CSF. Analysis of immunological outcomes showed activation of APCs and Ag-specific humoral and cellular immune responses which correlated with improved survival [10]; however, Sipuleucel-T only demonstrated a 4.1-month benefit to overall survival in three recent Phase III trials [11,12].

Lentivector (LV)-mediated transduction is an effective method for delivering TAAs to APCs. Antigens delivered by LV transductions are stably expressed and presented on Major Histocompatibility Complex (MHC) class I molecules [13,14]. Using LV-modified dendritic cells (DCs) engineered to overexpress prostate-specific antigen (PSA) and prostate-specific membrane antigen (PSMA), we have previously shown efficient induction of Ag-specific humoral and cell-mediated immunity in a murine model of PCa [15]. We further speculated that DCs transduced *in vivo* after direct LV injection would also be capable of eliciting effective Ag-specific immune responses against tumors. In fact, we demonstrated effective induction of human Ag-specific immunity and stabilization of established tumors after direct injection of LVs in a transgenic murine model of colorectal cancer [16].

To further explore the safety and clinical feasibility of direct LV vaccination, and to test this approach in an appropriate pre-clinical model, we developed a self-inactivating LV to immunize against PSA—a natural Ag that is upregulated in human PCa [17]. Indeed, we subcloned the rhesus (rh) orthologue of PSA for these studies. Firstly, direct injection of the novel LV we generated effectively elicited Ag-specific tumor protection in a murine rhPSA-tumor model. Secondly, for preclinical evaluation, LV/rhPSA was administered into rhesus macaques via direct intranodal injections. LV/rhPSA immunizations resulted in severe prostatitis and enlargement of the prostates which persisted for at least six months, while hematological parameters, plasma chemokine, and plasma cytokine levels remained within normal ranges for the duration of the study. These observations indicate that direct LV immunization against a natural TAA effectively overcomes self-tolerance to elicit a localized, tissue-specific immune response that may have therapeutic benefits for the treatment of cancer. Further, this direct injection approach involves an “off-the-shelf” product that, when modified to engineer expression of the human orthologue of PSA, could be given to patients, even in an out-patient setting.

## 2. Experimental Section

### 2.1. Lentivectors (LV)

LVs were produced as previously described [18]. Briefly, HEK293T cells were transiently transfected with the VSV-g-pseudotyping envelop plasmid (pMD.G), packaging plasmid (pCMVΔR8.91), and the transfer vector plasmid. The pHR′-cPPT-EF-GW.SIN transfer vector was used to generate LV/eGFP. Complimentary DNA sequences for rhPSA and rhCD25 were amplified from a rhesus macaque peripheral blood sample by PCR using primers for rhPSA (5′-GGAATTCGGCGCGCCACCATGTGGGTTCTGGTTGTCTTCCTCAC-3′ and 5′-GCTCTAGATCAGGGGTTTGCCATGATGGTG-3′) and rhCD25 (5′-GATATCGCCACCATGGATCCATACCTGCTCATGTGGG-3′ and 5′-TGCGGCCGCCTAGATTGTTCTTCTATTCTTCCTCTGTCTCCG-3′) and subcloned into pHR′-cPPT-EF-GW.SIN replacing eGFP to generate pHR′-cPPT-EF-rhPSA-IRES-rhCD25.SIN. Vector particles were concentrated by ultracentrifugation and suspended in serum-free RPMI-1640 medium. Functional vector titers were determined by transduction of HEK 293T cells using serially-diluted supernatant with analysis by flow cytometry for rhCD25 expression.

### 2.2. Flow Cytometry

Cells were stained with PE-conjugated anti-CD25 antibodies (BD Biosciences, San Jose, CA, USA) and analyzed for rhCD25 expression by flow cytometry.

### 2.3. Murine rhPSA Tumor Cell Lines

MOPC-315 murine (BALB/c) plasmacytoma cells [15] were cultured in DMEM supplemented with 10% FCS. MOPC-315 cells were transduced with LV/rhPSA/rhCD25 (described above) and sorted by flow cytometry into a high-expressing pool based on rhCD25 expression to yield the MOPC-315/rhPSA/rhCD25 cell line.

### 2.4. Western Blot

Whole cell lystates were normalized for protein content by use of the DC protein assay (Bio-Rad, Hercules, CA, USA) and resolved by SDS-PAGE. Proteins were stained using rabbit anti-human PSA antibodies (Dako Denmark A/S, Copenhagen, Denmark) and mouse anti-actin antibodies (EMD Millipore, Billerica, MA, USA).

### 2.5. Mice and Footpad Injections

All mouse procedures were performed under the University Health Network Animal Care Committee Animal Use Protocol 422.2 approved 6/13/05. BALB/c mice were purchased from The Jackson Laboratory (Bar Harbor, ME, USA). LV was administered by injection of 2 × 10^7^ IU of LV/eGFP or LV/rhPSA/rhCD25 to the mouse footpad at day 1 and day 14. For tumor challenge, mice were engrafted in subcutaneous (s.c.) tissue at day 21 with 1 × 10^6^ MOPC-315 and MOPC-315/rhPSA/rhCD25 tumor cells in the dorsal flank. Tumor growth was evaluated by caliper measurements until 11 days post-tumor challenge.

### 2.6. Rhesus Macaque Surgeries and LV Injections

All large animal procedures were performed under the University Health Network Animal Care Committee Animal Use Protocols 1512.4 approved 28 May 2009 and 2802.1 approved 13 August 2012. Six male rhesus macaques (*Macaca mulatta*) between 44–54 weeks old were housed individually within a negative-pressure regulated animal facility at the UHN. Animals were fasted for 12 h prior to the intranodal injection procedures. Animals were sedated with ketamine (10 mg/kg) and atropine (0.04 mg/kg) IM, and given buprenorphine (0.03 mg/kg) or gabapentin (0.06 mg/kg) IM for pre-emptive analgesia. Inhalational isofluorane was delivered by bag and mask for anesthesia and the animals were intubated, introduced with saline by cephalic I.V. line, and attached with monitoring equipment. After a sterile prep, 2–4 cm incisions were made over the lymph node (LN) basins. Individual nodes were identified by blunt and sharp dissection and injected with LV particles (5 to 25 µL per node, 1–2 × 10^8^ IU per animal) via a 27-gauge syringe. Hemostasis was confirmed and then the incisions were closed in two layers with a running subcuticular vicryl suture. Jackets were worn until the wounds healed (about two weeks). Post-operative treatments consisted of the broad-spectrum antibiotic enrofloxacin (5 mg/kg) P.O. twice daily for seven days, and buprenorphine (0.03 mg/kg) or gabapentin (0.06 mg/kg) I.M. twice daily as needed for analgesia. Detailed large animal care procedures employed are described in the Supplementary Methods.

### 2.7. Quantitative PCR

Quantitative PCR to detect proviral copies was performed as previously described [19]. Briefly, genomic DNA was extracted from tissue samples using the Gentra Puregene Blood Kit (Qiagen, Mississauga, ON, Canada) according to the manufacturer′s instructions. Quantitative PCR was carried out using a Rotor Gene RG300 system (Corbett Life Science, Cambridgeshire, UK) with primers for the WPRE (Woodchuck Hepatitus Virus posttranscriptional regulatory element). DNA extracted from a HEK293T cell line harboring one copy of LV provirus was used as standard [20].

### 2.8. Magnetic Resonance Imaging (MRI) for Prostate Volumes

MRI was used to determine the shapes and volumes of the macaque prostates at different time points. MRI scans were performed at the STTARR Facility (Toronto, ON, Canada). All procedures were performed following Standard Operating Procedures (SOPs) approved by the STTARR Facility and the UHN Animal Care Committee. Animals were subjected to volumetric T2-weighted imaging of the genitourinary region, to monitor the shapes and volumes of the prostate gland. Image-based quantification of the volume of the prostate gland was performed at baseline before LV injection and after LV injections. Volumetry scanning was performed by a 7-Tesla micro-MRI system (BioSpec 70/30 USR, Bruker BioSpin, Ettlingen, Germany), with a B-GA20S gradient coil and 15.5 cm inner diameter quadrature volume resonator. Imaging used a 3-D T2-weighted RARE imaging technique with 400-μm isotropic spatial resolution over a 80 × 80 × 36 mm field-of-view at 50 kHz readout bandwidth. Contrast determinants included an effective echo time of 86 ms, repetition time of 2200 ms, and a RARE factor of 18. The duration of the high resolution scan was 72 min. Prostate volumes were quantified by manual prescription of whole-gland contours on a slice-by-slice basis using MIPAV software (NIH, Bethesda, MD, USA).

### 2.9. Hematological Analyses

Heparinized whole blood was collected biweekly by venipuncture and analyzed at the UHN Animal Resource Center (Toronto, ON, Canada). General biochemistry was measured using a VetScan VS2 analyzer (Abaxis, Union City, CA, USA) and complete blood counts were measured using a Hemavet 950FS (Drew Scientific Inc., Waterbury, CT, USA).

### 2.10. Cytokine and Chemokine Measurements

Plasma samples were acquired from whole blood collected biweekly by venipuncture. Multiplex immunoassays were performed following the manufacturer’s protocols for the Th1/Th2 Cytokine Monkey 5-Plex kit (Life Technologies, Carlsbad, CA, USA) and Chemokine Monkey 5-Plex kit (Life Technologies, Carlsbad, CA, USA). Assays were analyzed on the Bio-Plex 200 multiplex array system (Bio-Rad, Hercules, CA, USA).

### 2.11. Pathology Analysis

Formalin-fixed and paraffin-embedded (FFPE) prostate tissue sections for pathology analysis were prepared at the UHN Pathology Research Program Laboratory (Toronto, ON, Canada). Briefly, prostate glands were collected from rhesus macaques at time of sacrifice six months post-LV injection and 1.5-μm sections were cut from FFPE tissue blocks with a Leica RM 2245 microtome (Leica Microsystems Inc, Buffalo Grove, IL, USA). Tissue sections were transferred onto microscope slides and deparaffinized using graded alcohol. Antigen retrieval was achieved by heat-induced epitope retrieval under pH 6.0 at 98°C followed by endogenous peroxidase blocking. Sections stained with primary antibodies directed against rhesus CD3, CD20, and CD25 (All from Abcam, Cambridge, UK).

### 2.12. Statistical Analyses

Statistical analyses between groups were performed using One-Way ANOVA with Tukey’s test on Prism 5 software (GraphPad Software Inc., La Jolla, CA, USA).

## 3. Results

### 3.1. LV/rhPSA/rhCD25 Vaccination Protects Mice from Relevant Tumor Challenge

**Figure 1:**
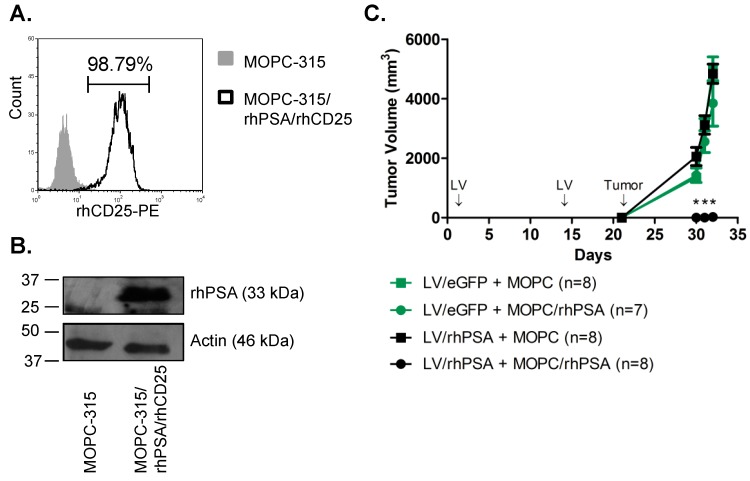
Effective antigen-specific tumor protection in immunized mice mediated by direct LV (lentivector) injections. MOPC-315 cells were transduced with LV/rhPSA/rhCD25 and sorted by flow cytometry for cells expressing rhCD25 to generate the stable MOPC-315/rhPSA/rhCD25 cell line. Expression of (**A**) rhCD25 and (**B**) rhPSA was confirmed by flow cytometry and Western blot analysis, respectively. (**C**) BALB/c mice received 2 × 10^7^ IU of LV/eGFP (green) or LV/rhPSA/rhCD25 (black) by footpad injections on days 1 and 14. MOPC-315 cells (squares) and stably transduced MOPC-315/rhPSA/rhCD25 cells (circles) were used to generate bilateral subcutaneous flank tumors 21 days post-immunization. Tumor volumes were determined by daily caliper measurements (mean volume ± SEM, * *p* < 0.05).

We generated a self-inactivating, bicistronic LV construct to transduce cells with the cDNAs for rhPSA and an rhCD25 cell-surface marker (LV/rhPSA/rhCD25; Figure S1). Engineered expression of rhCD25 as a cell-surface marker facilitates vector titering and tracking as we have shown before [15]. A LV/eGFP construct utilizing the same backbone was employed as the control. To demonstrate that immunization with LV/rhPSA/rhCD25 can stimulate an antigen-specific, tumor-protective response *in vivo*, we immunized mice following a regimen we previously established for direct LV injection [16]. A target murine tumor line expressing rhPSA was derived by transduction of MOPC-315 plasmacytoma cells followed by subsequent flow cytometric sorting for rhCD25-positive cells. Western blot and flow cytometric analyses confirmed the stable, high-level expression of both rhPSA and rhCD25, respectively, in the sorted population (MOPC-315/rhPSA/rhCD25; Figure 1A,B). BALB/c mice received direct footpad injections of LV/rhPSA/rhCD25 or control LV/eGFP on days 1 and 14, followed by tumor challenge with MOPC-315/rhPSA/rhCD25 cells or control MOPC-315 cells injected s.c. into the dorsal flanks on day 21 (Figure 1C). Large tumor growths developed within 11 days in LV/eGFP-immunized mice following tumor challenge from the parent MOPC-315 cells or MOPC-315/rhPSA/rhCD25 cells. In LV/rhPSA/rhCD25-immunized mice, only the non-modified parent MOPC-315 cells developed into tumors; growth of MOPC-315/rhPSA/rhCD25 cells was completely inhibited (Figure 1C). This data indicates that direct immunization with LV/rhPSA/rhCD25 produced a potent, antigen-specific immune response against rhPSA/rhCD25-expressing cells in mice, thus validating the efficacy of this recombinant LV and vaccination strategy.

### 3.2. Direct Lymph Node Immunization with LV/rhPSA/rhCD25 in Rhesus Macaques

**Figure 2:**
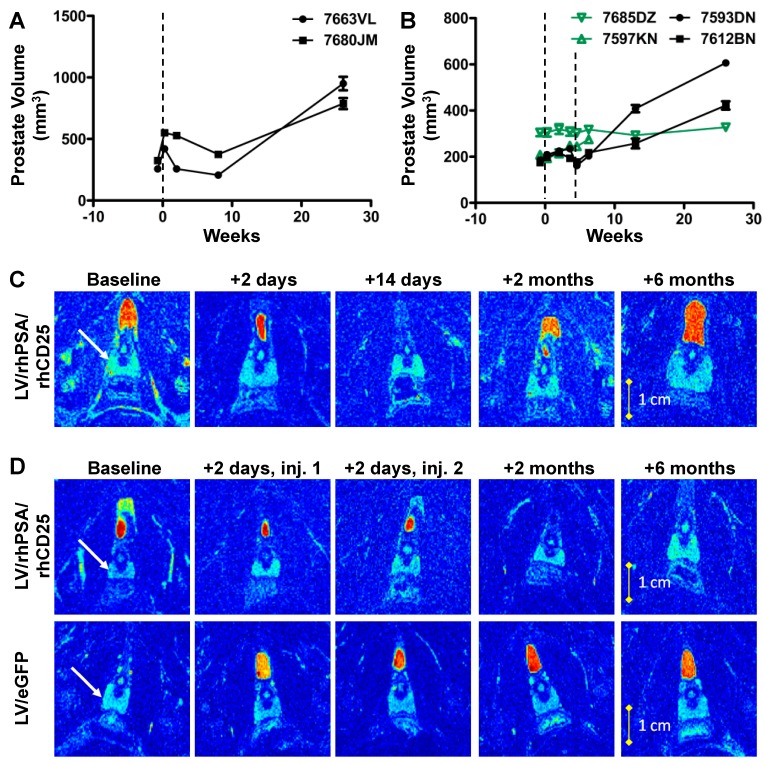
Enlarged prostate volumes in rhesus macaques following direct LV immunization. (**A**) Rhesus macaques were immunized with a single dose of 1–2 × 10^8^ IU of LV/rhPSA/rhCD25 by intranodal injections on day 0 (vertical dashed line). Prostate volumes were determined over time by MRI (mean volume ± SD). (**B**) Rhesus macaques were immunized with 0.5–1 × 10^8^ IU of LV/rhPSA/rhCD25 (black) or LV/eGFP (green) on days 0 and 28 (vertical dashed lines). Prostate volumes were determined over time by MRI (mean volume ± SD). Representative T2-weighted MR images of rhesus macaque prostates after receiving a (**C**) single-dose or a (**D**) prime-boost LV immunization through the region of the prostate of greatest cross-sectional area at each indicated time-point. White arrows in column 1 highlight the prostate gland (light blue).

To evaluate this approach in a pre-clinical context in a relevant large animal model, we proceeded to use this LV for direct immunization in rhesus macaques. In the first “proof-of-principle” study, two male macaques between the ages of 44–54 months with no history of immunological abnormalities or pre-existing infections received direct intranodal injections to deliver concentrated LV/rhPSA/rhCD25 particles under the capsule of the bilateral axillary, inguinal, and popliteal LNs. Each animal received a total LV dose of 1–2 × 10^8^ infectious units (IU) distributed among all sites that were successfully identified (9 to 13 LNs per animal). Both macaques exhibited clinical symptoms of severe prostatitis beginning on day 3, including enlarged prostates as determined by palpation. The recipient animals also displayed an unwillingness to present their midsections, as well as extended rubbing and scratching of their rectal area, weight loss, shivering, and a hunched posture. Animals were treated with buprenorphine (0.03 mg/kg) for pain relief as both animals also experienced muscle fasciculation lasting five days associated with acute pain in the prostate area. Reduced urine and feces production was observed in one of the animals and was resolved within a month. These animals then appeared healthy for the remainder of the study. Measurements by magnetic resonance imaging (MRI) of prostate volumes showed marked increases two days post-immunization that returned to pre-immunization volumes after two weeks and remained that volume when analyzed two months post-immunization (Figure 2A,C). Interestingly, the animals then again displayed dramatically enlarged prostates during an MRI follow-up six months post-immunization despite a lack of any other clinical symptoms (Figure 2A,C), thus suggesting that a specific, localized, and long-term response was elicited following such direct and targeted immunization.

### 3.3. Prime-Boost Administration of Smaller LV Doses Ameliorates Acute Responses

In a subsequent study, we refined the immunization method by implementing a prime-boost schema aimed at reducing vector burden at the time of administration while possibly generating more potent, long-lasting, cytotoxic T lymphocyte (CTL) responses [20,21]. Four macaques comprised this second cohort. Animals received either LV/rhPSA/rhCD25 or LV/eGFP as a control at the same total dose of 1–2 × 10^8^ IU as before, but administered as two equal doses of 0.5–1 × 10^8^ IU 28 days apart. In both the LV/rhPSA/rhCD25-treated group (*n* = 2) and the LV/eGFP group (*n* = 2) changes in prostate volume measurements by MRI were not observed immediately following vector administration (Figure 2B,D) nor did the animals display muscle fasciculation or other signs of pain. Nonetheless, animals were preemptively treated with gabapentin (0.06 mg/kg) in anticipation of treatment-associated pain. However, similar to the prior animals that received the immunization in a single dose, prostate sizes in animals administered with LV/rhPSA/rhCD25 in this prime-boost regimen were again significantly enlarged at the six-month assessment point (Figure 2B,D). In contrast, the animals that received LV/eGFP did not exhibit changes in prostate size at any point despite receiving an equivalent dose of LV particles (Figure 2B,D). Thus, by utilizing a prime-boost administration of LV, many of the acute complications were mitigated, while long-term, tissue-specific responses were still obtained.

### 3.4. Proviral Genomes Remained within the Site of Injection

It was of interest to next determine whether LV particles disseminated from the sites of LN injection in either macaque cohort. Qualitative PCR was performed using primers for the WPRE to detect proviral genomes in transduced cells from LNs and tissues were collected six months post-immunization (Table 1). Proviral genomes were detected in three of six animals, but only in the LNs. Among the animals vector-positive at six months, two received LV/eGFP immunizations and one received a single-dose LV/rhPSA/rhCD25 immunization. No animals were vector-positive after receiving the prime-boost immunization of LV/rhPSA/rhCD25. Taken together, these results demonstrate that direct LV administration led to long-term, tissue-specific responses regardless of vector persistence, thus suggesting outcomes were a result of immunological memory developed at the time of initial immunization as opposed to persistent restimulation from dormant vector-transduced cells.

### 3.5. Peripheral Blood Profiles Remained Normal after Direct LV Administration

**Figure 3:**
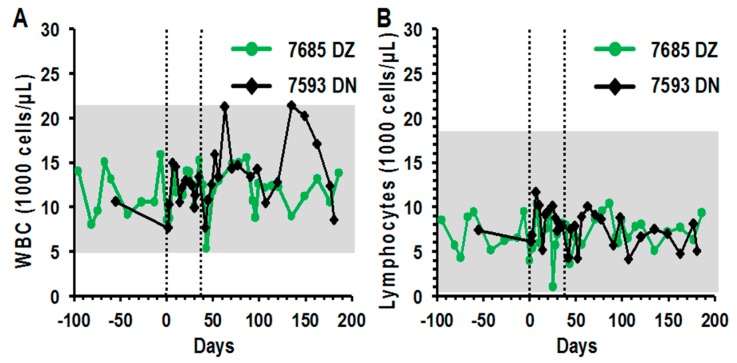

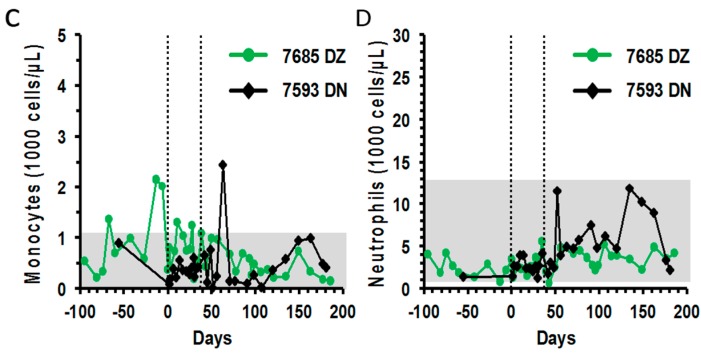
Direct LV-mediated immunizations did not perturb WBC differentials in rhesus macaques. Rhesus macaques were immunized with 0.5–1 × 10^8^ IU of LV/rhPSA/rhCD25 (black) or LV/eGFP (green) on days 0 and 28. Peripheral blood samples collected from rhesus macaques before and after immunization were analyzed for (**A**) white blood cell, (**B**) lymphocyte, (**C**) monocyte, and (**D**) neutrophil counts by Hemavet measurements. Vertical dotted lines indicate times of LV administration. Horizontal grey lines indicate normal ranges for the species.

**Figure 4:**
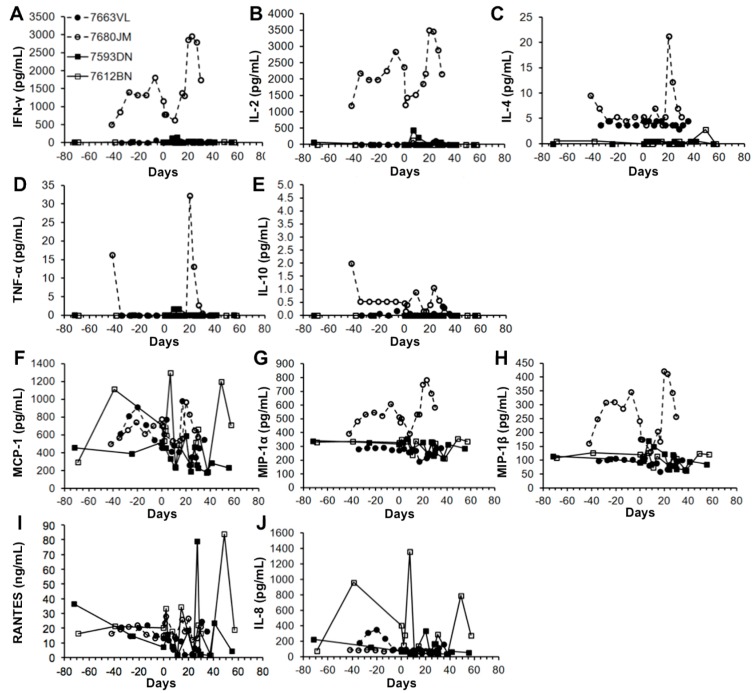
PSA immunization by LVs does not alter plasma cytokine and chemokine profiles. Rhesus macaques were immunized with either a single dose of 1–2 × 10^8^ IU of LV/rhPSA/rhCD25 by intranodal injections (circles) or two separate doses of 0.5–1 × 10^8^ IU of LV/rhPSA/rhCD25 (squares) on days 0 and 28. Plasma samples collected over time before and after immunization were analyzed by multiplex immunoassay for (**A**) IFN-γ, (**B**) IL-2, (**C**) IL-4, (**D**) TNF-α, (**E**) IL-10, (**F**) MCP-1, (**G**) MIP-1α, (**H**) MIP-1β, (**I**) RANTES, and (**J**) IL-8.

Peripheral blood was collected biweekly to monitor cell counts and blood chemistry along with plasma cytokine and chemokine levels. In both LV/rhPSA/rhCD25- and LV/eGFP-injected groups, total white blood cell, lymphocyte, monocyte, and neutrophil counts remained within normal ranges for the duration of the study (Figure 3A–D). Red blood cell compositions and blood chemistry were also within the normal ranges for both groups for the duration of the study (Figure S2A–D, Figure S3A,B). Cytokine and chemokine levels were analyzed from biweekly plasma samples by multiplex immunoassays. In both the single-dose and prime-boost administration groups, cytokine (IFN-γ, IL-2, IL-4, IL-10 and TNF-α) and chemokine (MCP-1, MIP-1α, MIP-1β, RANTES and IL-8) levels remained at baseline before and after treatment (Figure 4A–J). These analyses indicate that severe systemic perturbations did not occur following direct LV-mediated immunizations into the LNs.

## 4. Discussion

Successful immunotherapy for cancer requires effective antigen uptake, meaningful antigen presentation, and potent and persistent effector responses. A plethora of contextual and tumor-based immune-suppressive factors often limit such events from occurring optimally [4]; consequently, immunotherapeutic strategies have been developed to facilitate key events in generating anti-tumor responses. In this study, LVs encoding a species-specific TAA were administered directly to the lymph nodes—the hub for immune cell interaction—rather than using LVs as an auxiliary reagent in *ex vivo* immune cell modification schemas. LVs are an appropriate delivery schema in this context given the relative safety of later generation constructs and their propensity for long-term expression in transduced cells. Injection of LV particles to the LNs directly delivers the transgene payload at a high concentration to potent APCs such as resident DCs that potently induce Th1 and Th2 responses [22]. Furthermore, Ag presentation kinetics are improved compared to other routes of administration as diffusion effects from systemic injections and subsequent migration distances are vastly reduced and/or eliminated.

To date we have demonstrated effective antigen-specific protection from tumor challenge and control of established tumor growth by direct LV immunizations in two murine models targeting distinct tumor antigens [16]. Here we employ this direct injection approach in an important translational model that will allow administration and evaluation of the treatment in relevant clinical scales. We validated this principle in non-human primates using direct intranodal LV injections to immunize against a natural self-antigen that is also an orthologue to a known human TAA. Immune responses against self-antigens are tightly regulated, and self-reactive clones are usually deleted or anergized at various stages of development as a natural mechanism to prevent autoimmune responses. This becomes an obstacle for generating potent tumor-specific responses as most TAAs are dysregulated or mutated forms of self-antigens. After receiving a single dose of LV/rhPSA/rhCD25 we observed acute and chronic prostatitis. This acute prostatitis was accompanied, unfortunately, with clinical manifestations of pain and distress that required treatment with analgesics. A second cohort of macaques was treated by administering the vector with two separate doses 28 days apart and only injecting half the number of LNs each time. Analgesics were preemptively administered in the second cohort. This method of delivery reduced stress from invasive surgical procedures and vector burden at the time of administration and effectively mitigated acute adverse reactions while preserving long-term prostate enlargement effects. Furthermore, delivering the immunization in a prime-boost schema is also known to generate higher frequencies of high avidity CTLs [20,21].

The mechanism leading to prostate inflammation and enlargement in this rhesus macaque model following direct LV injection has not been fully elucidated. Optimization of the timing for sacrifice/tissue collection was difficult; animal numbers and resources were limited and our primary interest was to examine long-term safety with this novel approach. Immunohistochemical (IHC) analyses for CD3^+^ and CD20^+^ cells (Figure S4A,B) and *in vitro* restimulation of prostate-infiltrating T cells did not show statistical differences between LV/rhPSA/rhCD25- and LV/eGFP-prime-boost-immunized prostate samples collected at the end of the study. This could be a consequence of the delay in analysis. That said, a trend towards increased levels of CD3 staining and decreased CD20 staining was observed in prostates of animals that received LV/rhPSA/rhCD25 injections, indicating a shift toward a T-mediated response (Figure S4A,B), as is expected following prime-boost immunization [20,21]. Interestingly, the trend toward increased levels of CD3 staining was accompanied by an inclination towards increased staining for the CD25 marker on transduced cells following LV/rhPSA/rhCD25 prime-boost immunization (Figure S4C).

The current observations suggest that the prostatitis observed after the animals received the LV/rhPSA/rhCD25 immunization was an antigen-specific reaction as LV/eGFP-immunized macaques did not experience any changes in the prostate. This conclusion is further supported by the Ag-specific tumor immunity observed in murine tumor models receiving immunizations of LV/rhPSA/CD25 (Figure 1) and LV/huCEA [16]. Findings from this study indicate that LV immunizations administered by intranodal injections were able to overcome self-tolerance to stimulate an immune response towards the self-antigen, rhPSA. Furthermore, long-term prostate-specific responses persisted for at least six months and are desirable in cancer immunotherapy to prevent tumor relapse events.

Of key importance to clinical translation is the safety of this novel immunization strategy. Complete blood counts and blood chemistry remained within normal ranges and cytokine and chemokine levels remained at pre-immunization levels for all recipients throughout the course of the study. One recipient animal had transient elevations in neutrophil counts, but the timing suggested they were in response to an unrelated infection. Other than inflammation of the target organ following rhPSA immunization, animals remained healthy for the duration of the study, reinforcing the enhanced safety of direct LV immunization.

It is intriguing to speculate that our choice of rhesus CD25 as a cell surface marker to facilitate detection of transduced cells may have actually contributed to the strong response we observed. It has been shown that CD25 overexpression on its own in human dendritic cells mediates trans-signaling events that lead to activation of T cells [23,24]. In addition, a soluble form of CD25 is present in culture supernatants of activated mononuclear cells and in normal human serum [25]. Though the functions of soluble CD25 are largely unknown, it may regulate immune signaling. It is not known if overexpressed CD25 from LV-infected cells could be processed *in vivo* and form soluble CD25, whose functions are unclear. Further studies need to be done to investigate the potential role of macaque CD25 in immune activation and its contribution, if any, to the responses observed in this study.

An exhaustive multi-organ analysis was carried out to detect vector proviral DNA. Of all the tissues tested, vector proviral DNA was detected only in LNs. Animals that received single-dose LV/rhPSA/rhCD25 and prime-boost LV/eGFP were positive but animals that received prime-boost LV/rhPSA/rhCD25 were not. Residual cells harboring proviral genomic DNA at that site could be LN stromal cells with slow turnover rates, LN resident APCs, or even memory lymphocytes that have returned to the LNs after performing their effector functions, the presence of which is favorable for long-lasting immunological memory [26,27]. The absence of transduced cells even in the LNs of animals receiving prime-boost LV/rhPSA/rhCD25 is perplexing, though we speculate that we were near the level of detection in our assays given the longer time point chosen. As well, functionally transduced cells could have even been targeted by the induced immune response against rhPSA following treatment.

Intranodal delivery of immunotherapeutics, such as DCs [28], DNA vaccines [29], and canarypox virus vectors [30], has shown to improve CTL responses in cancer patients. Usage of LVs in immunotherapy has primarily been limited to *ex vivo* modification of therapeutic cells, such as antigen-loading of APCs [31] or receptor modulation of effector T cells [1]. While both strategies have demonstrated some recent clinical success, the requirement for personalized patient care and personalized cell manipulation limits widespread implementation of such therapies. The LV immunotherapy described in this study can easily be adapted for any known TAA and produced in an “off-the-shelf” format; however, the choice of antigen becomes of crucial importance. Desirable candidate antigens should present with evidence of self-reactive lymphocytes and limited expression in normal tissues. As with other targeted immunotherapies, off-target cytotoxicity could conceivably lead to serious consequences [32].

In conclusion, we have demonstrated for the first time in a clinically relevant, large animal model that an intranodal LV immunization strategy as described in this study may be an effective method to stimulate immune responses against an otherwise tolerized self-antigen. With modern LV production technology, mass production of “off-the-shelf” LV vaccines is possible for any known TAA, making direct LV immunization into LNs a promising modality for development of a universal cancer therapy.

## Figures and Tables

**Table 1:** Multi-tissue quantitative PCR analyses of LV proviral DNA six months post immunization.

Organ	LV/rhPSA/rhCD25 Single Injection		LV/rhPSA/rhCD25 Prime-Boost		LV/eGFP Prime-Boost	
	7680JM	7663VL	7612BN	7593DN	7597KN	7685DZ
Heart	−	−	−	−	N.D.	N.D.
Liver	−	−	−	−	N.D.	N.D.
Thymus	−	−	−	−	N.D.	N.D.
Lung	−	−	−	−	N.D.	N.D.
Testicles	−	−	−	−	N.D.	N.D.
Spleen	−	−	−	−	N.D.	N.D.
Mesenteric LN	−	−	−	−	+	+
Right Inguinal LN	−	−	−	−	+	−
Left Axillary LN	−	−	−	−	−	−
Right Axillary LN	−	−	−	−	−	−
Left Popliteal LN	+	−	−	−	−	−
Left Inguinal LN	−	−	−	−	+	−

N.D. = No data.

## Supplementary Materials

Supplementary materials can be found at http://www.mdpi.com/2227-9059/4/1/6/s1.
