# Supplementary material for: Direct Lymph Node Vaccination of Lentivector/Prostate-Specific Antigen is Safe and Generates Tissue-Specific Responses in Rhesus Macaques
Source: Biomedicines. 2016 Feb 19;4(1):6. doi: 10.3390/biomedicines4010006 (PMC5344243; doi:10.3390/biomedicines4010006)
Supplement: Supplementary file 1 [file biomedicines-04-00006-s001.pdf]

Supplementary Materials: Direct Lymph Node Vaccination of Lentivector/Prostate-Specific Antigen is Safe and Generates Tissue-Specific Responses in Rhesus Macaques

Supplementary Methods

Rhesus Macaque Animal Care

All large animal procedures were performed under protocols approved by the UHN Animal Care Committee. All animals tested serologically negative for SIV, STLV, simian retrovirus, and herpes B virus and were routinely monitored for other infectious pathogens. Six male rhesus macaques (*Macaca mulatta*) between 44–54 weeks old were housed individually within a negative-pressure regulated animal facility at the UHN. A low-residue diet was given to the animals prior to the surgery and they were fasted for 12 h prior to the procedure itself. Vascular access ports were implanted four to six weeks before treatment. Animals were sedated with ketamine (10 mg/kg) and atropine (0.04 mg/kg) IM, and given buprenorphine IM (0.03 mg/kg) for pre-emptive analgesia. Inhalational isofluorane was delivered by bag and mask for anesthesia. Animals were intubated and an IV line was inserted into the saphenous vein. Full monitoring including BP, HR, temp, EKG, capnography, and O_2 saturation were performed by UHN veterinarians. The injection procedure was performed with the animal supine on a 3M Bair Hugger warming blanket (Arizant Healthcare, St. Paul, MN, USA) to maintain body temperature. The animal was shaved from neck to groin and washed with a hibitane scrub (Zoetis Canada Inc, Kirkland, QC, Canada). The entire skin was prepped with Betadine (Purdue Products L.C., Stamford, CT, USA) three times. Two to four cm incisions were made over the LN basins. Individual nodes were identified by blunt and sharp dissection and injected with LV particles (5 to 25 μL per node, 1–2 × 10^8 IU per animal) via a 27-gauge syringe. Hemostasis was confirmed and then the incisions were closed in two layers with a running subcuticular vicryl suture. Bacitracin was then applied to the surgical site. Jackets were worn until the wounds healed (about two weeks). Post-operative treatments consisted of the broad spectrum antibiotic enrofloxacin (5 mg/kg P.O.) twice daily for seven days, and buprenorphine (0.03 mg/kg I.M.) twice daily as needed for analgesia. Prior to MRI procedures, the macaques were intubated, oriented in a supine position within a plexiglass form, and advanced into the system feet-first. An anesthetic regime for MRI imaging matched that of the surgical preparation, except that MR-compatible devices were used for physiologic monitoring (non-invasive blood pressure monitoring and capnography, ECG, temperature).

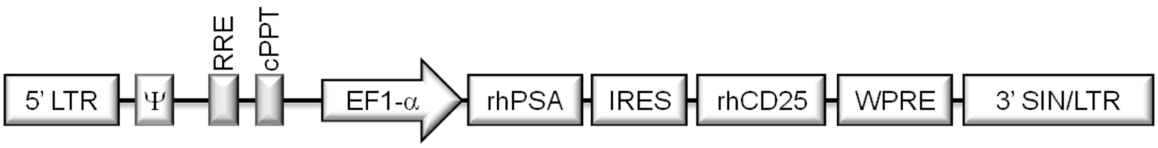

Figure S1. Bicistronic LV/rhPSA/rhCD25 vector design. Schematic representation of LV/rhPSA/rhCD25. Expression of rhPSA and rhCD25 is regulated by the EF1-*α* promoter with an IRES sequence to allow translation of rhCD25. LTR, long-terminal repeat; Ψ, packaging sequence; RRE, rev response element; cPPT, central polypurine tract; EF1-*α*, EF1-*α* promoter; IRES, encephalomyocarditis virus internal ribosomal entry site; WPRE, woodchuck hepatitis virus posttranscriptional regulatory element; SIN/LTR: self-inactivating LTR.

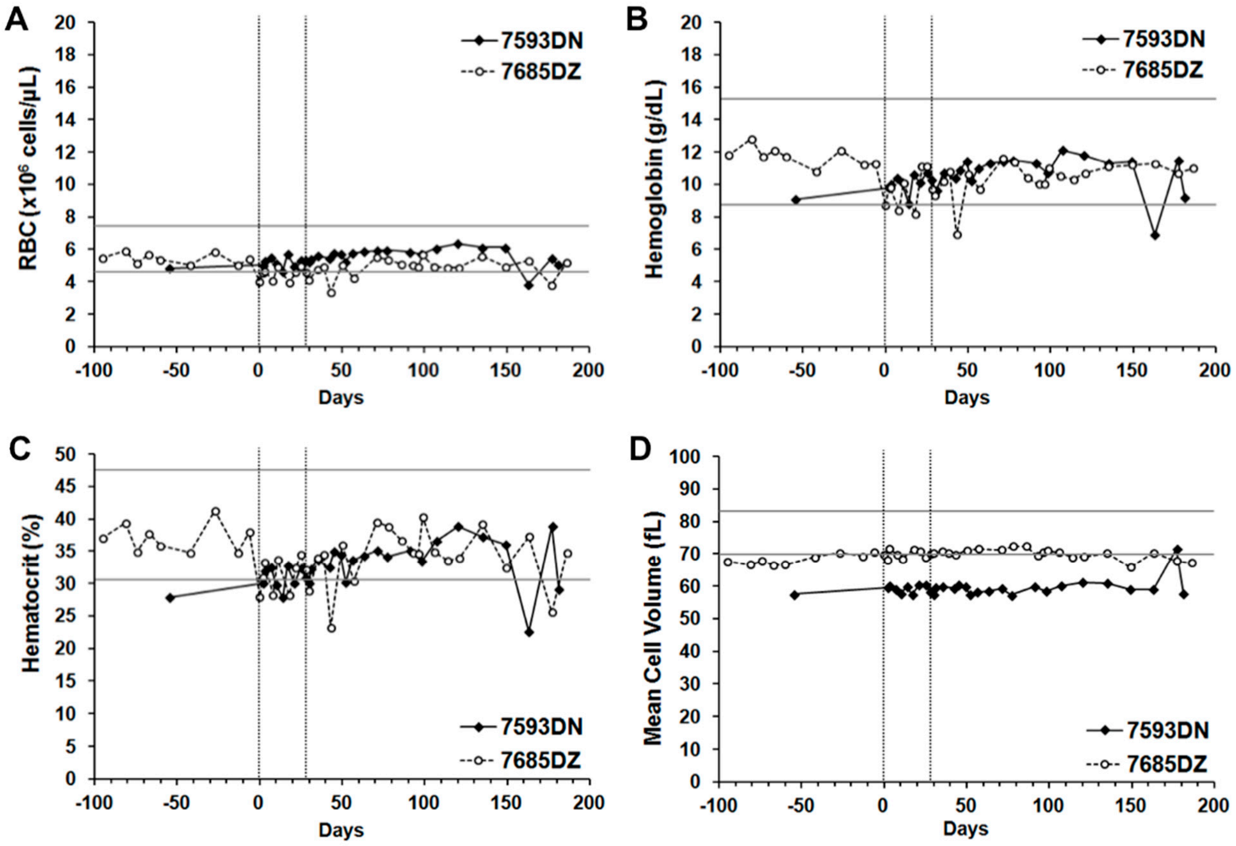

Figure S2. Direct LV-mediated immunizations did not alter RBC composition in rhesus macaques. Rhesus macaques were immunized with 0.5–1 × 10^8 IU of LV/rhPSA/rhCD25 (solid line) or LV/eGFP (dashed line) on days 0 and 28. Peripheral blood samples collected from rhesus macaques before and after immunization were analyzed for (**A**) red blood cell (RBC) counts, (**B**) hemoglobin, (**C**) hematocrit, and (**D**) mean cell volume by Hemavet measurements. Vertical dotted lines indicate times of LV administration. Horizontal grey lines indicate normal ranges for the species.

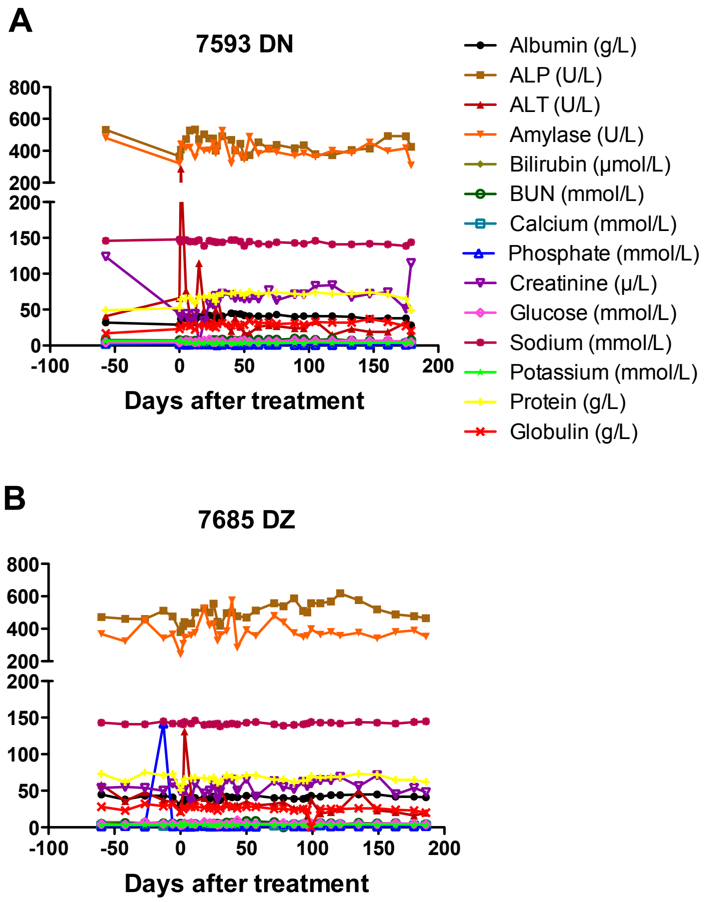

Figure S3. Peripheral blood chemistry was not affected by direct LV-mediated immunizations. Rhesus macaques were immunized with 0.5–1 × 10^8 IU of (**A**) LV/rhPSA/rhCD25 (7593 DN) or (**B**) LV/eGFP (7685 DZ) on days 0 and 28. Hematologic chemistry and electrolytes were analyzed on peripheral blood samples collected from rhesus macaques before and after immunization.

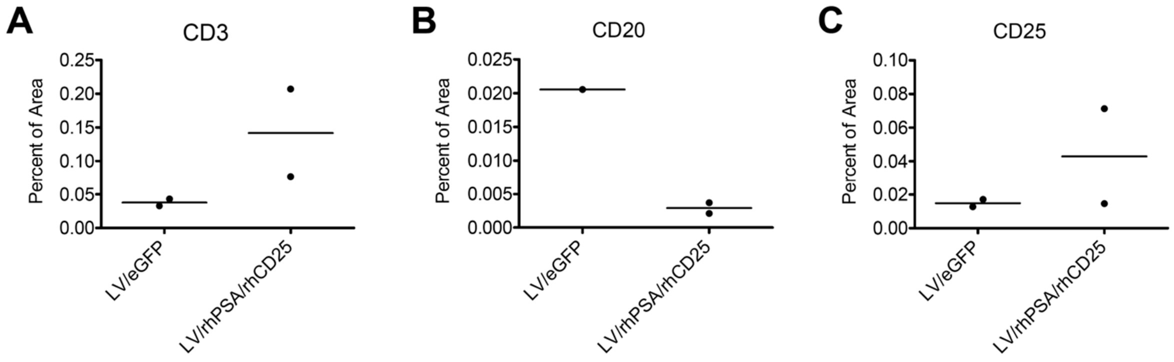

Figure S4. Immunohistological analyses of prostate tissues reveal trend in increased rhCD3 and rhCD25 cells**.** Rhesus macaques were immunized with 0.5–1 × 10^8 IU of LV/rhPSA/rhCD25 or LV/eGFP on days 0 and 28. Prostate tissue samples were collected six months post-immunization and were stained by immunohistochemistry for (**A**) rhCD3, (**B**) rhCD20, and (**C**) rhCD25.
